# Lipid Mediators in Skin Inflammation: Updates and Current Views

**DOI:** 10.1155/2010/398926

**Published:** 2010-10-21

**Authors:** Giuseppe Valacchi, Chiara De Luca, Philip W. Wertz

**Affiliations:** ^1^Department of Biomedical Sciences, University of Siena, 53100 Siena, USA; ^2^Department of Food and Nutrition, Research Institute of Human Ecology, Kyung Hee University, Seoul 130-701, Republic of Korea; ^3^Laboratory of Tissue Engineering and Skin Pathophysiology, Istituto Dermopatico dell'Immacolata (IDI IRCCS), 00167 Rome, Italy; ^4^University of Iowa Dows Institute for Dental Research, Iowa City, Iowa 52242, USA

This special issue is dedicated to those lipid mediators playing roles in skin inflammation, a topic in continuous evolution, due to relevant findings connecting the alterations of lipid metabolism occurring systemically and at the skin level. Currently, one of the main hopes is to find clinically relevant biomarkers of disease susceptibility and progression. It is also important to identify molecules with therapeutic potential for the main inflammatory pathologies with significant socioeconomic impact involving the skin. Among these psoriasis, atopic dermatitis, or diabetic skin ulcer, as well as less severe dermatologic conditions like acne or premature aging, seriously affect patient quality of life and psychological scores.

The skin is indeed a very peculiar organ, displaying biochemical and immunological features specific for a compartment continuously exposed to external and endogenous stimuli. Skin lipids, with their unique biochemical composition, provide protection from environmental stressors although the formation of bioactive compounds such as 4-hydroxynonenal, oxysterols, and oxidized phospholipids can be a consequence of exposure to these stressors. These compounds derived mainly from the oxidation of the skin lipids are essential in the regulation of skin and mucosal tissue inflammation, since they are able to trigger, sustain, or terminate cutaneous inflammatory processes. Their function in the modulation of ROS, RNS, cytokine, and chemokine production and release, in DNA and protein oxidation, in gene expression, in the regulation of antioxidant and detoxifying enzymes, is under extensive investigation. Their control may therefore well represent a feasible tool for clinical interventions aiming at the correct modulation of skin physiological functions for possible disease prevention.

In line with these concepts, the articles contributing to this issue provide new information or review current *status* of research on the etiological role of lipid molecules and their metabolites in acute or chronic inflammatory states of the skin compartment, induced by endogenous diseases or environmental factors.

Innovative approaches like postgenomics and lipidomics are presented in the work of D. Braconi et al., as powerful tools waiting for more extensive application in a deeper investigation on skin pathophysiological processes. Systemic and topical lipid oxidation is a key mechanism involved in chronic inflammatory skin diseases, among which psoriasis, acne, atopic, and seborrheic dermatitis are the most compelling examples, as presented by S. Pastore et al. and A. Pietrzak et al. More extensively, the dysregulation of the strict homeostatic systems active in the skin to maintain the redox balance and counteract abnormal oxidative stress in chronic inflammatory skin diseases is widely analysed, with special attention to how this highly controlled mechanism is affected by therapeutic intervention.

Among skin lipids, the surface hydrolipidic film has been studied for a long time, due to the very peculiar composition of the sebaceous lipids and their specific contribution to skin protection and isolation. The work by C. De Luca et al. presents an overview of the current literature on the oxidation of squalene and of other degradable components of human sebum and the functions that these compounds play in mediating the effects of UV light and other environmental physical, chemical, and microbiological stressors on the skin layers. In addition, the role of oxidative stress in dermatological diseases such as psoriasis and atopic dermatitis as a trigger for such pathologies and a possible therapeutic tool is discussed in the work by S. Pastore et al. and A. Pietrzak et al. The specific role of sebum-derived lipid mediators in the aetiology of acne is updated by E. Camera et al., focussing on the latest advances concerning their possible interference with biochemical patterns of sebocyte differentiation and sebogenesis.

Lipid modifications induced by resident and pathogenic microbial flora, affecting inflammatory *status* of the skin, also receive proper attention in the issue. Locally, a deficit of immune response possibly due to loss of specific subsets of dendritic cells may result in sustained inflammatory events, secondary to repeated skin infections, as in the case of Darier's Disease as reported by C. Miracco et al.

Finally, extensive space is devoted to possible clinical intervention tools, based on chemical or physical principles directed at the prevention and suppression of excessive lipid oxidation. The toxicity of skin lipid reactive mediators is addressed with particular attention to the active principles for systemic and topical application (C. De Luca et al.), aimed at the prevention not only of sunburn but also of local and systemic immunological impairment, resulting in increased risk of photo-carcinogenesis. On the other side, skin lipid oxidation products seem to bear promising implications as effective agents for the phototherapy of skin hyperproliferative states.

Genomic approaches are proposed by J. V. Gruber et al. for the individuation of specific effects of active natural plant phenolics on genes implicated in the main skin pathophysiological processes, in view of the recent appraisal of plant extracts as inhibitors of skin lipoperoxidation and proinflammatory cytokines release. Alternative physical treatments are reviewed, including the use of therapeutically active ozonated derivatives (V. Travagli et al.), delivered through an unsaturated lipid vehicle generating reactive mediators *in situ*, for more efficient treatment of recurrent skin infections resistant to topical antibiotics. The feasibility of dietary supplementation in the control of skin inflammatory processes is well exemplified in the work by N.-Y. Park et al. where SKH1 mice fed with conjugated linoleic acid showed an enhancement of the early stage of cutaneous wound healing, as a result of the modulation of oxidative stress and inflammatory response.

In addition, the possible deleterious effects of nonsteroidal anti-inflammatory drugs or selective cyclooxygenase-2 inhibitors on the fragile balance of proinflammatory mediators in the course of wound healing are summarized by W.-H. Su et al., contributing to the search for new therapeutic tools possibly proving less harmful to the local skin cytokine environment.

*Giuseppe Valacchi* *Giuseppe Valacchi* *Chiara De Luca* *Chiara De Luca* *Philip W. Wertz* *Philip W. Wertz*

